# Common carotid artery intima-media thickness is useful for diagnosis of the acute stage of Kawasaki disease

**DOI:** 10.1186/1471-2431-14-98

**Published:** 2014-04-10

**Authors:** Ting-Hsin Wu, Hsuan-Chang Kuo, You-Lin Tain, Kuan-Miao Lin, Ho-Chang Kuo, Shao-Ju Chien

**Affiliations:** 1 Department of Pediatrics, Kaohsiung Chang Gung Memorial Hospital and College of Medicine, Chang Gung University, 123 Ta-Pei Road, Niaosung, Kaohsiung, Taiwan

**Keywords:** Kawasaki disease, Common carotid artery, Intima-media thickness, Acute stage

## Abstract

**Background:**

This study aimed to investigate intima-media thickness (IMT) of the common carotid arteries in children with acute Kawasaki disease (KD).

**Methods:**

Between 2009 and 2011, patients fulfilling the criteria for KD, including a fever lasting >5 days, were prospectively enrolled in this study. Laboratory data, echocardiography, and IMT were measured and compared with matched controls.

**Results:**

A total of 70 common carotid IMTs were measured in 35 children. We studied 21 patients aged 3–60 months old with acute KD and 14 febrile patients aged 3–194 months old with acute infection and similar characteristics to those of KD patients. Children with KD had a significantly higher IMT compared with the controls (0.550 ± 0.081 mm vs. 0.483 ± 0.046 mm, *P* = 0.01).

**Conclusions:**

IMT during the acute stage of KD is increased, suggesting that IMT could be a useful diagnostic tool in the early diagnosis of KD.

## Background

Kawasaki disease (KD) is an acute systemic vasculitis that mainly affects medium-sized arteries in multiple systems and primarily occurs in children under the age of 5 years. Diagnosis of KD is clinically made using non-specific diagnostic guidelines, including prolonged fever, conjunctivitis, diffuse mucosal inflammation, polymorphous skin rashes, indurative edema of the hands and feet associated with peeling of the fingertips, and non-suppurative lymphadenopathy [1,2]. There is currently no definitive laboratory test available for diagnosis of KD. Diagnosis of KD is further complicated because the above-listed clinical criteria may be transient. In addition, the constellation of principle physical findings of KD may also vary over time or are not obvious either in the early stages of disease or in cases of incomplete KD. An expedited diagnosis of this disease is crucial to administer appropriate therapy and to potentially limit the development of coronary artery lesions [1,2]. The most serious complication in KD is coronary aneurysm due to severe inflammation and vasculitis of the coronary arteries, especially when treatment is delayed [3-7]. Treatment with intravenous immunoglobulin within the first 10 days after onset of KD is highly effective for the acute phase of this illness and considerably reduces the prevalence of coronary artery complications [1,3,7-10]. Therefore, early recognition and prompt treatment of KD are crucial.

Measurement of intima-media thickness (IMT) of common carotid arteries is a widely used and validated noninvasive imaging technique for the assessment of early structural changes in the arterial wall. Inflammation of vessel walls precedes morphological changes and is believed to be the initial step in many rheumatic diseases, including Takayasu’s arteritis, [11,12] systemic lupus erythematosus, [13] rheumatoid arthritis, [14] Behçet’s disease, [15] and ankylosing spondylitis [16,17]. Only a few published studies, some of them controversial, have investigated the development of an increased IMT in the common carotid artery in patients diagnosed with KD. Previous studies have reported that in long-term follow up, carotid artery IMT is greater in patients with KD, and patients with coronary artery involvement after KD have the largest IMT [18,19].

The purpose of this study was to investigate IMT in the acute stage of KD and to determine the level of vascular involvement. This study describes the ultrasound findings of carotid IMT in patients with active KD to evaluate its utility as a marker of disease activity and to elucidate the role of carotid IMT in the early diagnosis of KD.

## Methods

Between 2009 and 2011, 21 patients, aged 3–60 months old, treated at the Kaohsiung Chang Gung Children’s Hospital and fulfilling the criteria for KD (more than four principle criteria and a fever lasting >5 days) were prospectively enrolled in this study as previously described in detail [9,20,21]. Fourteen febrile patients, aged 3–194 months old, with acute infections and similar characteristics to those of KD patients underwent the same imaging studies (two-dimensional echocardiography and B-mode ultrasound) for a heart murmur survey, and were used as the control group. The control group comprised patients who had pneumococcal pneumonia (n = 5), salmonella enterocolitis (n = 4), or acute pyelonephritis (n = 5). Patients whose symptoms did not fit the criteria for KD, had an acute fever for <5 days, or in cases where data were incomplete were excluded. Children with known heart disease or traditional risk factors of atherosclerosis, such as hypertension, diabetes mellitus, a family history of premature congestive heart disease, and obesity (body mass index [BMI] >95th percentile for the age-specific reference group), were also excluded.

In addition to performing echocardiography and ultrasonography of the common carotid arteries, all included patients underwent clinical evaluation, and their age, sex, weight, height, (BMI), pulse, and blood pressure were recorded. Blood pressure was the mean of two consecutive measurements obtained with the patient in a seated position after resting for >5 min. Laboratory tests routinely performed included a complete blood cell count, differential white blood cell count, and C-reactive protein (CRP; reference range, ≤5 mg/L) measurement.

### Two-dimensional echocardiography and B-mode ultrasonography

All patients with KD underwent two-dimensional echocardiography of the coronary artery and high-resolution B-mode ultrasonography of the bilateral common carotid arteries to measure IMT prior to intravenous immunoglobulin treatment. All of the children in the control group underwent the same examinations as KD patients during acute infections. The left coronary artery was measured midway between the ostium and the bifurcation of the circumflex artery and the left anterior descending coronary artery in the parasternal short-axis view. The proximal right coronary artery was obtained 3 to 5 mm distal to its origin in the parasternal short-axis view. Coronary artery dimensions were measured and z-scores were calculated from the formula derived from Dallaire et al. [22] in 2011 using regression with the square root of body surface area because of the differences in age and BMI between the two groups. We chose this method because this equation yielded pediatric z-scores with an appropriate normal distribution across the entire range of body surface area on the basis of a large number of infants, children, and adolescents. The z-scores beyond the normal limits (cutoff of Z = ±2) were considered abnormal. The incidence of abnormal coronary arteries was also analyzed.

**Figure 1:**
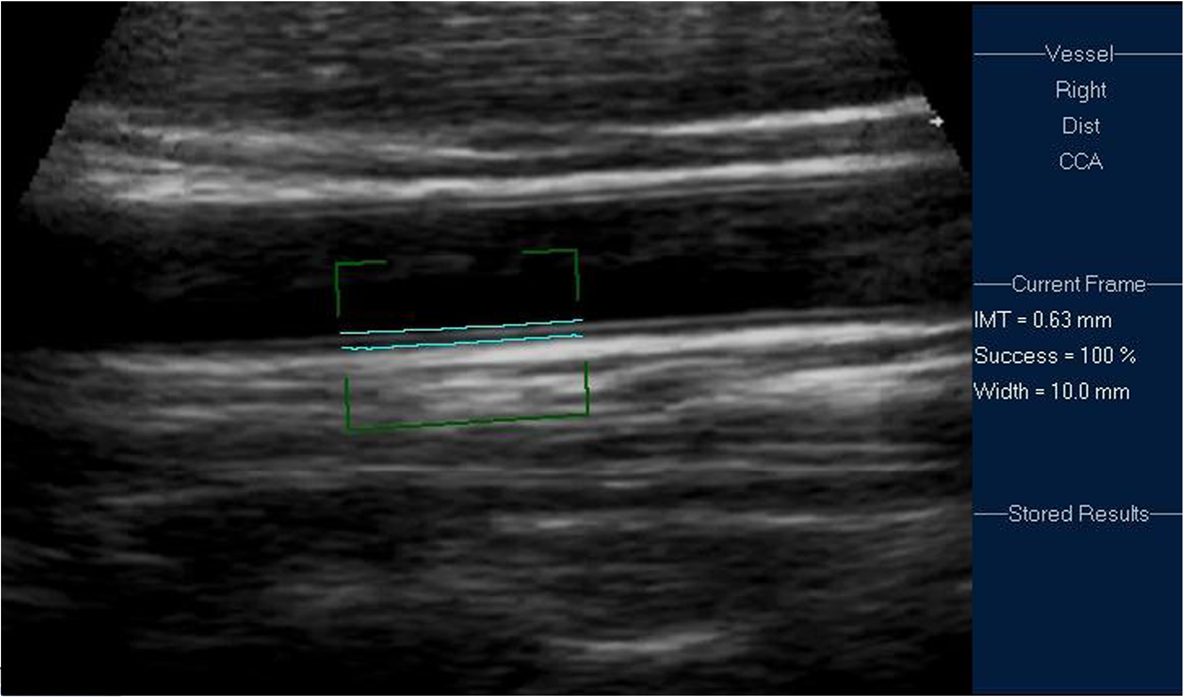
Measurement of carotid IMT in Qlab.

A single, experienced pediatric cardiologist who was blinded to the diagnoses performed all of the carotid ultrasound assessments. The carotid ultrasonographic studies were performed under standardized conditions with the patients in the supine position for at least 10 min in a quiet room prior to examination. For data acquisition, high-resolution ultrasound equipment (SONOS 7500; Phillips Medical Systems, Andover, MA) with an 11-MHz linear array probe was used. All studies were performed according to a standardized scanning protocol for the right and left common carotid arteries [21-24]. During the examination, all children were in the supine position with their heads turned slightly to the side. The transducer was manipulated so that the near and far walls of the common carotid arteries were parallel to the transducer footprint and the lumen diameter was maximum in the longitudinal plane. The entire carotid proximal common carotid artery was observed approximately 1.5 cm before the bifurcation. We used the distance between the leading edges of the luminal-intimal interface and the medial-adventitial interface for the measurement of IMT. IMT was measured during end diastole as determined by the R wave on an electrocardiogram. We also scrolled through the cine loop and measured IMT at the arteries’ largest diameter in two infants in the KD group because they were not cooperative during monitoring of the electrocardiogram. All images were stored digitally and subsequently analyzed offline. We used Qlab Software (Philips, Germany) to analyze the IMT distance automatically at 64 points within a segment of 10 mm. The value reported by this software was the arithmetic mean IMT. The IMT echo was assessed and measured with calipers within a standardized higher resolution zoom. We chose the image of the best quality with the clearest edge (Figure 1), which was always obtained with the interfaces oriented perpendicular to the ultrasound beam. This image was acquired, temporarily stored in the cine loop, and consecutively zoomed in Qlab when the measurements were performed. Manual overreading of border detection during computed analysis was performed for all images. Each measurement was accompanied by a “success rate”, which was the percentage of the intima-media within the region of interest that was accurately measured. We only used the measured values that had a success rate of ≥95%. The use of standard automatic procedures for IMT measurements limits the variability related to human error and allows comparability between studies. Our experienced pediatric cardiologist had performed carotid ultrasound for several years. For reproducibility of IMT measurements, we calculated the intraobserver coefficient of variation, which was 4.1%.

This study protocol was approved by the Institutional Review Board of Chang Gung Memorial Hospital, Taiwan and was performed in accordance with the ethical standards laid down in the 1964 Declaration of Helsinki. Informed consent was obtained from all parents prior to the children’s inclusion in the study.

### Statistical analysis

All continuous data are expressed as mean ± standard deviation of the mean and minimum and maximum values. We used non-parametric statistical procedures because of the small number of data. Categorical variables were analyzed with Fisher’s exact test and the chi-square test when appropriate. We also performed multivariate regression analysis using IMT as the dependent variable, and age, BMI, and KD as independent variables to assess whether different factors affected carotid IMT. All analyses were performed using the Statistical Package for Social Sciences, version 14.0 for Windows XP (SPSS, Inc., Chicago, USA).

## Results

### Demographic data and coronary artery diameter

Patient demographics are shown in Tables 1 and 2. Controls and KD patients had similar characteristics, with no significant differences in age, sex, body size, or blood pressure. Because there was no routine oral sedation for the heart murmur survey, clear edges of IMT were easier to obtain from older children who were more cooperative for carotid ultrasound. Although the age in the control group was older than that in the KD group, this was not significantly different. There were no significant differences in weight, height, BMI, or systolic and diastolic blood pressure between the two groups. There was also no significant difference in CRP levels or white blood cell counts between the groups. Platelet counts were significantly higher in KD patients than in controls. There was no significant difference in coronary artery diameter between the two groups. However, the incidence of z-scores beyond the normal limit (cutoff of Z = ±2) of the left coronary artery and left anterior descending coronary artery in KD patients was significantly higher than that in the control group.

**Table 1:** Demographic data and blood pressure of patients with KD and febrile controls

**Characteristic**	**Patients with KD (n = 21)**	**Controls (n = 14)**	***p *** value*
Male sex (%)	71.4%	71.4%	1.00
Mean age (months)	21.79 ± 17.91	52.18 ± 56.52	0.13
Body weight (kg)	11.37 ± 3.93	19.17 ± 14.50	0.08
Body height (cm)	82.32 ± 14.91	100.54 ± 29.91	0.21
BMI (kg/m2)	16.45 ± 1.50	18.08 ± 2.64	0.14
Systolic BP (mmHg)	103 ± 16	112 ± 14	0.35
Diastolic BP (mmHg)	63 ± 13	71 ± 11	0.09

Values are mean ± SD or %.

BMI, body mass index; BP, blood pressure; KD, Kawasaki disease.

*Mann–Whitney U test.

**Table 2:** Inflammation markers, echocardiographic parameters, and IMT values in patients with KD and febrile controls

**Characteristic**	**Patients with KD (n = 21)**	**Controls (n = 14)**	***p *** value*
LCA (mm)	2.79 ± 0.77	2.51 ± 0.56	0.35
z-score >2 or < -2	52.4% (n = 11)	7.1% (n = 1)	0.01
LAD (mm)	2.30 ± 0.90	1.90 ± 0.35	0.40
z-score >2 or < -2	47.6% (n = 10)	0	0.00
LCX (mm)	1.64 ± 0.63	1.45 ± 0.17	0.57
z-score >2 or < -2	9.5% (n = 2)	0	0.51
RCA (mm)	2.57 ± 1.24	2.17 ± 0.54	0.59
z-score >2 or < -2	17.1% (n = 6)	0	0.06
LVEF (%)	65 ± 7	65 ± 6	0.96
Total WBC (×1000/mm^3^)	16.12 ± 7.01	15.13 ± 6.95	0.69
Platelets (×1000/mm^3^)	487.5 ± 198.0	352.5 ± 161.1	0.01
CRP (mg/L)	110.4 ± 91.5	148.6 ± 89.6	0.17
IMT (mm)	0.550 ± 0.08	0.483 ± 0.05	0.01

Values are mean ± SD or %.

LCA, left coronary artery; LAD, left anterior descending coronary artery; LCX, left circumflex coronary artery; RCA, right coronary artery; LVEF, left ventricular ejection fraction; WBC, white blood cells; CRP, C-reactive protein; IMT, intima-media thickness.

*Mann–Whitney U test.

### Mean carotid IMT

**Figure 2:**
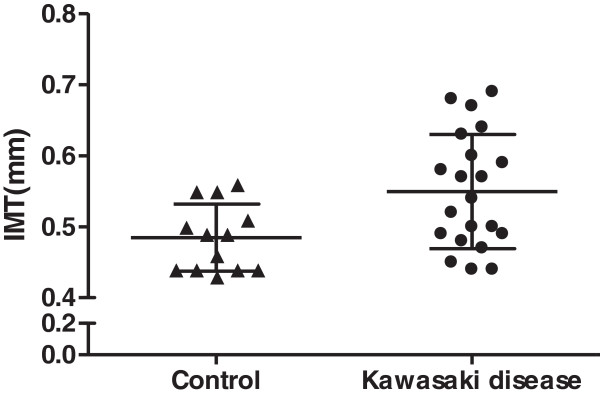
Distribution of IMT measurements. Bars show mean ± SD.

Measurement of carotid IMT by Qlab software, accompanied by the IMT within a segment of 10 mm and its 100% success rate on the right side, are shown in Figure 1. The distribution of IMT is shown in Figure 2. Mean carotid IMT in KD patients (0.550 ± 0.081 mm; range, 0.44–0.69 mm), was significantly higher than that in the febrile control group (0.483 mm ± 0.046 mm; range, 0.43–0.56 mm; *P* = 0.01) (Table 2). Even if age and BMI were not significantly different between KD patients and controls, the difference between the two groups was highly relevant. We performed multiple linear regression analysis using IMT measurement as the dependent variable to exclude the confounding factors of age and BMI. In multivariate analysis, only KD was consistently associated with intima-medial thickening (Table 3).

**Table 3:** Multivariate regression model of IMT measurements with KD, age, and BMI

	**IMT measurements**		
**Variables**	**Regression coefficient**	**Standard error**	***p *** **value**
KD	0.072	0.028	0.017
Age	0.000	0.000	0.465
BMI	0.005	0.006	0.471

IMT, intima-media thickness; BMI, body mass index; KD, Kawasaki disease.

## Discussion

Our study showed that carotid IMT was higher in the acute stage of KD compared with other acute infections. Although there was no significant difference in age between the two groups, higher IMT of KD patients may still be due to age. Age-associated changes in IMT in young children have not yet been fully examined. Pauciullo *et al.*[23] reported that the mean carotid IMT of healthy children aged 6 ± 3 years was 0.39 ± 0.03 mm and Ishizu *et al.*[24] reported that it was 0.44 ± 0.05 mm among children aged between 5 to 14 years old. An age-related increase in carotid IMT with an annual 0.009 mm increase in healthy children has been reported to reflect the physiological growing process [24]. Theoretically, in our study, IMT in KD patients should have been much lower than that in the control group because of their age. However, we found that IMT was significantly higher in KD patients who were younger than the control group. Therefore, the difference between groups is due to the underlying disease rather than the selection bias of age. Furthermore, we performed multivariate regression analysis, which showed that IMT was the only independent factor for KD. All of the other factors lost their statistical influence on the IMT thickening. These preliminary results suggest that an increased IMT of the carotid artery in any acutely ill, febrile child should raise suspicion for KD. Therefore, carotid ultrasonography is an important diagnostic tool in the early diagnosis of KD if an increased IMT is shown in conjunction with additional clinical signs.

Over the last few years, a large number of studies have emphasized the fundamental role of ultrasonography in the early diagnosis of vasculitis in adult patients. These studies demonstrated abnormal ultrasonographic changes in the acute phase of Takayasu disease, [11,12,24,25]. Mediterranean fever, [26] temporal arteritis, [27] and Behçet’s disease [15]. These diseases are considered to have early structural vascular alterations and atherosclerosis because of their ongoing subclinical inflammation. In these diseases, IMT was used to assess blood vessels and to help with early detection. Intravascular ultrasound studies in patients with Takayasu showed thickening and altered echogenicity of the arterial wall [24]. Most authors believe that these changes are a result of acute dysfunction of the endothelium and inflammation of the vascular wall rather than chronic proliferative and fibrotic changes of atherosclerosis.

To the best of the authors’ knowledge, no specific IMT studies of common carotid arteries have been performed to help diagnose KD. The present study attempted to distinguish KD from other infection diseases on the basis of IMT. We also attempted to determine the level of vascular involvement by evaluating the role of IMT in the diagnosis of KD. In contrast to the combination of cellular and fibrous proliferation, which accumulates during long-term follow-up after KD, increased IMT in the acute stage is entirely attributed to acute inflammation [28]. Impairment of endothelial function precedes morphological changes and is believed to be the initial step in the development of many other inflammatory rheumatic diseases. Dysfunction of the endothelium and the presence of macrophages and activated lymphocytes within the vessel wall lead to thickening of the intima and media of the vessel wall of large- and medium-sized muscular arteries [29]. High-resolution B-mode ultrasonography is useful in showing the same characteristic features of homogenous hyperechogenicity of the thickened arterial wall [11,29].

One noteworthy limitation of this study is that the ultrasonographic preliminary results were not correlated with pathological findings. Pathological correlation may help physicians further understand the causes of increased wall thickness. This limitation occurred because in KD, biopsy is restricted to opportunities, such as intraoperative biopsy during vascular reconstruction surgery. Another limitation of our study is that our KD patients did not have any coronary artery abnormalities or aneurysms. During our short-term follow up, we did not find a trend of persistent increased IMT as in previous studies [17]. These different preliminary results between studies may be because KD patients enrolled in our study did not have any considerable coronary artery involvement. This transient IMT phenomenon is similar to their transient coronary dilation. Among KD children without marked coronary artery abnormalities (as recruited in our study), intima-medial thickening during the acute phase is probably a transient, subclinical phenomenon without long-term sequelae on atherosclerotic risk. Considering the small number of cases included in our study, these results are primarily preliminary and further studies including a larger number of patients are warranted. IMT can hopefully be used to reliably identify children most at risk for severe disease.

## Conclusions

In children with laboratory preliminary results indicative of vasculitis or suspicion of KD, but who do not fulfill the criteria of KD, IMT could be an additional diagnostic tool used to determine the level of vascular involvement. This will help achieve an early diagnosis of KD and expedite establishment of an appropriate therapy.

## Abbreviations

BMI: Body mass index; IMT: Intima-media thickness; KD: Kawasaki disease.

## Competing interests

The authors declare that they have no competing interests.

## Authors’ contributions

SJC and TSW conceived of and participated in the design of the study, and drafted the manuscript. YLT and KML participated in the design of the study and performed the statistical analysis. HsCK and HoCK participated in coordination of the study and helped to draft the manuscript. All authors read and approved the final manuscript.

## Pre-publication history

The pre-publication history for this paper can be accessed here:

http://www.biomedcentral.com/1471-2431/14/98/prepub

## References

[B1] Newburger JW Takahashi M Gerber MA Gewitz MH Tani LY Burns JC Shulman ST Bolger AF Ferrieri P Baltimore RS Wilson WR Baddour LM Levison ME Pallasch TJ Falace DA Taubert KA Diagnosis, treatment, and long-term management of Kawasaki disease: a statement for health professionals from the Committee on Rheumatic Fever, Endocarditis and Kawasaki Disease, Council on Cardiovascular Disease in the Young, American Heart Association Circulation 2004 110 2747 2771 10.1161/01.CIR.0000145143.19711.78 15505111

[B2] Kuo HC Chang WC Genetic polymorphisms in Kawasaki disease Acta Pharmacol Sin 2011 32 1193 1198 10.1038/aps.2011.93 21892198 PMC4010074

[B3] Wang CL Wu YT Liu CA Kuo HC Yang KD Kawasaki disease: infection, immunity and genetics Pediatr Infect Dis J 2005 24 998 1004 10.1097/01.inf.0000183786.70519.fa 16282937

[B4] Fukazawa R Long-term prognosis of Kawasaki disease: increased cardiovascular risk? Curr Opin Pediatr 2010 22 587 592 20717036 10.1097/MOP.0b013e32833e12f7

[B5] Benseler SM McCrindle BW Silverman ED Tyrrell PN Wong J Yeung RS Infections and Kawasaki disease: implications for coronary artery outcome Pediatrics 2005 116 e760 e766 10.1542/peds.2005-0559 16322132

[B6] Burns JC Glode MP Kawasaki syndrome Lancet 2004 364 533 544 10.1016/S0140-6736(04)16814-1 15302199

[B7] Senzaki H Long-term outcome of Kawasaki disease Circulation 2008 118 2763 2772 10.1161/CIRCULATIONAHA.107.749515 19106401

[B8] Kuo HC Yang KD Chang WC Ger LP Hsieh KS Kawasaki disease: an update on diagnosis and treatment Pediatr Neonatol 2012 53 4 11 10.1016/j.pedneo.2011.11.003 22348488

[B9] Kuo HC Yang KD Juo SH Liang CD Chen WC Wang YS Lee CH Hsi E Yu HR Woon PY Lin IC Huang CF Hwang DY Lee CP Lin LY Chang WP Chang WC ITPKC single nucleotide polymorphism associated with the Kawasaki disease in a Taiwanese population PLoS ONE 2011 6 e17370 10.1371/journal.pone.0017370 21533171 PMC3077380

[B10] Kuo HC Liang CD Yu HR Wang CL Lin IC Liu CA Chang JC Lee CP Chang WC Yang KD CTLA-4, position 49 A/G polymorphism associated with coronary artery lesions in Kawasaki disease J Clin Immunol 2011 31 240 244 10.1007/s10875-010-9484-4 21082224

[B11] Schmidt WA Nerenheim A Seipelt E Poehls C Gromnica-Ihle E Diagnosis of early Takayasu arteritis with sonography Rheumatology (Oxford) 2002 41 496 502 10.1093/rheumatology/41.5.496 12011371

[B12] Lima DS Sato EI Lima VC Miranda F Jr Hatta FH Brachial endothelial function is impaired in patients with systemic lupus erythematosus J Rheumatol 2002 29 292 297 11842823

[B13] Van Doornum S McColl G Jenkins A Green DJ Wicks IP Screening for atherosclerosis in patients with rheumatoid arthritis: comparison of two in vivo tests of vascular function Arthritis Rheum 2003 48 72 80 10.1002/art.10735 12528106

[B14] Oflaz H Mercanoglu F Karaman O Kamali S Erer B Genchellac H Pamukcu B Umman S Inanc M Gul A Impaired endothelium-dependent flow-mediated dilation in Behcet’s disease: more prominent endothelial dysfunction in patients with vascular involvement Int J Clin Prac 2005 59 777 781 10.1111/j.1742-1241.2005.00477.x 15963203

[B15] Poredos P Intima-media thickness: indicator of cardiovascular risk and measure of the extent of atherosclerosis Vasc Med 2004 9 46 54 10.1191/1358863x04vm514ra 15230488

[B16] Sari I Okan T Akar S Cece H Altay C Secil M Birlik M Onen F Akkoc N Impaired endothelial function in patients with ankylosing spondylitis Rheumatology (Oxford) 2006 45 283 286 16204374 10.1093/rheumatology/kei145

[B17] Dalla Pozza R Bechtold S Urschel S Kozlik-Feldmann R Netz H Subclinical atherosclerosis, but normal autonomic function after Kawasaki disease J Pediatr 2007 151 239 243 10.1016/j.jpeds.2007.03.057 17719930

[B18] Cheung YF Wong SJ Ho MH Relationship between carotid intima-media thickness and arterial stiffness in children after Kawasaki disease Arch Dis Childhood 2007 92 43 47 10.1136/adc.2006.096628 16820386 PMC2083125

[B19] de Groot E Hovingh GK Wiegman A Duriez P Smit AJ Fruchart JC Kastelein JJ Measurement of arterial wall thickness as a surrogate marker for atherosclerosis Circulation 2004 109 23 Suppl 1 III33 III38 15198964 10.1161/01.CIR.0000131516.65699.ba

[B20] Lee YC Kuo HC Chang JS Chang LY Huang LM Chen MR Liang CD Chi H Huang FY Lee ML Huang YC Hwang B Chiu NC Hwang KP Lee PC Chang LC Liu YM Chen YJ Chen CH Chen YT Tsai FJ Wu JY Two new susceptibility loci for Kawasaki disease identified through genome-wide association analysis Nat Genet 2012 44 522 525 10.1038/ng.2227 22446961

[B21] Kuo HC Yang KD Liang CD Bong CN Yu HR Wang L Wang CL The relationship of eosinophilia to intravenous immunoglobulin treatment failure in Kawasaki disease Pediatr Allergy Immunol 2007 18 354 359 10.1111/j.1399-3038.2007.00516.x 17584314

[B22] Dallaire F Dahdah N New equations and a critical appraisal of coronary artery Z scores in healthy children J Am Soc Echocardiography 2011 24 60 74 10.1016/j.echo.2010.10.004 21074965

[B23] Pauciullo P Iannuzzi A Sartorio R Irace C Covetti G Di Costanzo A Rubba P Increased intima-media thickness of the common carotid artery in hypercholesterolemic children Arterioscler Thromb 1994 14 1075 1079 10.1161/01.ATV.14.7.1075 8018662

[B24] Ishizu T Ishimitsu T Yanagi H Seo Y Obara K Moriyama N Watanabe S Yamaguchi I Effect of age on carotid arterial intima-media thickness in childhood Heart Vessels 2004 19 189 195 15278393 10.1007/s00380-004-0766-8

[B25] Seth S Goyal NK Jagia P Gulati G Karthikeyan G Sharma S Talwar KK Carotid intima-medial thickness as a marker of disease activity in Takayasu’s arteritis Int J Cardiol 2006 108 385 390 10.1016/j.ijcard.2005.05.033 15970340

[B26] Park SH Chung JW Lee JW Han MH Park JH Carotid artery involvement in Takayasu’s arteritis: evaluation of the activity by ultrasonography J Ultrasound Med 2001 20 371 378 11316316 10.7863/jum.2001.20.4.371

[B27] Peru H Altun B Dogan M Kara F Elmaci AM Oran B The evaluation of carotid intima-media thickness in children with familial Mediterranean fever Clin Rheumatol 2008 27 689 694 10.1007/s10067-007-0764-1 17926078

[B28] Slyper AH Clinical review 168: what vascular ultrasound testing has revealed about pediatric atherogenesis, and a potential clinical role for ultrasound in pediatric risk assessment J Clin Endocrinol Metab 2004 89 3089 3095 10.1210/jc.2003-030644 15240574

[B29] Karassa FB Matsagas MI Schmidt WA Ioannidis JP Meta-analysis: test performance of ultrasonography for giant-cell arteritis Ann Intern Med 2005 142 359 369 10.7326/0003-4819-142-5-200503010-00011 15738455

